# An embedded longitudinal multi-faceted qualitative evaluation of a complex cluster randomized controlled trial aiming to reduce clinically important errors in medicines management in general practice

**DOI:** 10.1186/1745-6215-13-78

**Published:** 2012-06-08

**Authors:** Kathrin M Cresswell, Stacey Sadler, Sarah Rodgers, Anthony Avery, Judith Cantrill, Scott A Murray, Aziz Sheikh

**Affiliations:** 1 eHealth Research Group, Centre for Population Health Sciences, The University of Edinburgh, Scotland, UK; 2 NHS Nottinghamshire County, Nottingham, UK; 3 Collaboration for Leadership in Applied Health Research and Care, University of Nottingham, Nottingham, UK; 4 Community Health Sciences, University of Nottingham, Nottingham, UK; 5 Drug Usage and Pharmacy Practice Group, School of Pharmacy and Pharmaceutical Sciences, University of Manchester, Manchester, UK

**Keywords:** Qualitative evaluation, Randomized controlled trial, Pharmacist intervention, Primary care

## Abstract

**Background:**

There is a need to shed light on the pathways through which complex interventions mediate their effects in order to enable critical reflection on their transferability. We sought to explore and understand key stakeholder accounts of the acceptability, likely impact and strategies for optimizing and rolling-out a successful pharmacist-led information technology-enabled (PINCER) intervention, which substantially reduced the risk of clinically important errors in medicines management in primary care.

**Methods:**

Data were collected at two geographical locations in central England through a combination of one-to-one longitudinal semi-structured telephone interviews (one at the beginning of the trial and another when the trial was well underway), relevant documents, and focus group discussions following delivery of the PINCER intervention. Participants included PINCER pharmacists, general practice staff, researchers involved in the running of the trial, and primary care trust staff. PINCER pharmacists were interviewed at three different time-points during the delivery of the PINCER intervention. Analysis was thematic with diffusion of innovation theory providing a theoretical framework.

**Results:**

We conducted 52 semi-structured telephone interviews and six focus group discussions with 30 additional participants. In addition, documentary data were collected from six pharmacist diaries, along with notes from four meetings of the PINCER pharmacists and feedback meetings from 34 practices. Key findings that helped to explain the success of the PINCER intervention included the perceived importance of focusing on prescribing errors to all stakeholders, and the credibility and appropriateness of a pharmacist-led intervention to address these shortcomings. Central to this was the face-to-face contact and relationship building between pharmacists and a range of practice staff, and pharmacists’ explicitly designated role as a change agent. However, important concerns were identified about the likely sustainability of this new model of delivering care, in the absence of an appropriate support network for pharmacists and career development pathways.

**Conclusions:**

This embedded qualitative inquiry has helped to understand the complex organizational and social environment in which the trial was undertaken and the PINCER intervention was delivered. The longitudinal element has given insight into the dynamic changes and developments over time. Medication errors and ways to address these are high on stakeholders’ agendas. Our results further indicate that pharmacists were, because of their professional standing and skill-set, able to engage with the complex general practice environment and able to identify and manage many clinically important errors in medicines management. The transferability of the PINCER intervention approach, both in relation to other prescribing errors and to other practices, is likely to be high.

## Background

Medication errors (particularly those pertaining to prescribing and monitoring) are responsible for considerable morbidity and, in some cases, mortality; the majority of these are however believed to be preventable [1-3]. In the absence of a robust evidence base on how to reduce the high disease burden resulting from prescribing errors, there is an urgent need to investigate new ways of enhancing prescribing safety and reducing the risk of iatrogenic harm.

The highest rates of medication errors tend to be found in patients taking multiple medications and also in relation to certain drugs that are frequently associated with preventable morbidity [4-9]. Reasons for these errors have been studied in-depth over recent years and this work has shown that both individual factors (for example, knowledge about medication and slips in attention) as well as organizational factors (for example, communication, work environment, workload, training, supervision) are key contributory factors [10-14].

As most prescribing activity takes place in the general practice (GP) environment, primary care has been the focus of recent efforts to address the issue of medication errors [15]. In this context, there is suggestive evidence that pharmacist-led interventions in primary care may reduce hospital admissions, but to date there has been no confirmatory evidence from robust randomized controlled trials in this setting [16].

Building on a body of descriptive, qualitative and pilot work, we undertook a large complex intervention trial of a pharmacist-led information technology-enabled (PINCER) intervention compared with Simple feedback to reduce clinically important medication error rates. Details of the PINCER trial design and findings have been reported elsewhere [17,18], but in essence, data were obtained from ‘at-risk’ patients from ten defined outcome measures, identified through searches of GP computer systems. The trial outcome measures are summarized in Table 1. The interventions were delivered in 72 practices in two locations in England. General practices were centrally randomized to computer-generated Simple feedback on at-risk patients (control arm) or the PINCER intervention comprising feedback, educational outreach and dedicated support (intervention arm). We considered it unethical to provide no feedback to the control arm practices and provided these practices with computer-generated feedback on at-risk patients, with which they were free to intervene as they saw fit. All practices therefore received at least some form of intervention.

**Table 1:** Outcome measures in the PINCER Trial

	
**Primary outcome measures**	The proportion of patients in each practice:
	1. With a history of peptic ulcer being prescribed non-selective non-steroidal anti-inflammatory drugs
	2. With a history of asthma being prescribed beta-blockers
	3. Aged 75 years and older receiving long-term prescriptions for angiotensin-converting enzyme inhibitors or loop diuretics without a recorded assessment of renal function and electrolytes in the preceding 15 months.
	4. Proportions of women with a past medical history of venous or arterial thrombosis who have been prescribed the combined oral contraceptive pill
**Secondary outcome measures**	5. Patients receiving methotrexate for at least three months who have not had a recorded full blood count and/or liver function test within the previous three months
	6. Patients receiving warfarin for at least three months who have not had a recorded check of their international normalized ratio within the previous 12 weeks
	7. Patients receiving lithium for at least three months who have not had a recorded check of their lithium levels within the previous three months
	8. Patients receiving amiodarone for at least six months who have not had a thyroid function test within the previous six months
	9. Patients receiving prescriptions of methotrexate without instructions that the drug should be taken weekly
	10. Patients receiving prescriptions of amiodarone for at least one month who were receiving a dose of more than 200 mg per day.

In the PINCER intervention, pharmacists were allocated to work in practices for a period of three days per week for up to 12 weeks. Pharmacists’ work involved delivering computer-generated feedback to practice staff obtained through electronic searches of individual practice systems conducted by other members of the research team. Pharmacists used educational outreach and root cause analysis techniques to identify potential causes of clinically important errors in medicines management, and to assist practices in making changes to patients’ medication. Simple feedback practices were given computer-generated written feedback on clinically important errors in medicines management and were asked to make changes to identified patients’ medication themselves in the 12-week intervention period. Quantitative data were collected and analyzed at baseline and six and 12 months post-intervention. Although the six month juncture represented the main trial endpoint, we were also interested in studying the sustained impact of the PINCER intervention, hence our *a priori* decision to also assess outcomes at 12 months post-intervention.

At both six and 12 months follow-up, patients in the PINCER intervention practices were significantly less likely to have a prescribing problem or a monitoring problem (measured as composite outcome measures). These findings are discussed at length in our related paper [18]; the main results are summarized in Table 2.

**Table 2:** Main findings from the trial in relation to primary outcome measures

**Outcome measure**	**Six months follow-up**	**12 months follow-up**
Patients with a history of peptic ulcer being prescribed non-selective NSAIDs (nonsteroidal anti-inflammatory drugs)	Significant reduction in error rates in PINCER intervention practices when compared to Simple feedback practices (OR 0.58, 95% CI 0.38, 0.89)	Reduced error rates in PINCER intervention practices when compared to Simple feedback practices but no longer significant (OR 0.91, 95% CI 0.59, 1.39)
Patients with a history of asthma being prescribed beta-blockers	Significant reduction in error rates in PINCER intervention practices when compared to Simple feedback practices (OR 0.73, 95% CI 0.58, 0.91)	Significant reduction in error rates in PINCER intervention practices when compared to Simple feedback practices (OR 0.78, 95% CI 0.63, 0.97)
Patients aged 75 years and older receiving long-term prescriptions for ACE inhibitors or loop diuretics without a recorded assessment of renal function and electrolytes in the preceding 15 months.	Significant reduction in error rates in PINCER intervention practices when compared to Simple feedback practices (OR 0.51, 95% CI 0.34, 0.78)	Significant reduction in error rates in PINCER intervention practices when compared to Simple feedback practices (OR 0.63, 95% CI 0.41, 0.95)

CI, confidence interval; OR, odds ratio.

The value of qualitative methods in complementing complex randomized controlled trials by gaining an insight into the causal pathways involved is increasingly recognized in health services research [19-26]. For example, the Medical Research Council (MRC) highlights the value of mixed method approaches to evaluating complex interventions, with a qualitative component running parallel to a main trial investigating processes and issues surrounding the potential transferability of results to other settings [22,23]. Similarly, Bradley and colleagues have recently argued that qualitative methods can help to explain quantitative findings and therefore facilitate the design of more effective future interventions [24]. A central component of a qualitative evaluation of complex interventions is a focus on the experiences and viewpoints of different stakeholders over time.

In line with this thinking, we conducted an embedded longitudinal multi-faceted qualitative inquiry. We hoped that this would help us interpret the trial findings by providing an insight into the social and organizational context in which the trial was being conducted, shedding light on the likely causal pathways through which the pharmacist-led approach mediated its effects, and by assessing the likely transferability of the PINCER intervention [27-29]. Furthermore, we hoped to build on existing literature that has, while recognizing the value of qualitative methods in complementing complex randomized controlled trials, typically failed to combine both quantitative and qualitative findings into an integrated and meaningful whole [27,28].

## Methods

### Sampling

In order to obtain a sufficiently rounded understanding of the contextual factors surrounding the delivery of the PINCER intervention, a variety of key stakeholders were purposively sampled from the two different geographical locations in which the main trial was delivered [30,31].

The sampling strategy included approaching PINCER pharmacists to identify practice staff from PINCER intervention practices, while participants from Simple feedback practices were recruited with the help of the research team who had conducted the recruitment visits (in order to compare the different approaches). This typically involved initially approaching the practice manager, who then suggested further participants within their practice. A range of practice staff was interviewed including GPs, practice managers, nurses and administrative staff from both PINCER intervention and Simple feedback control arms. Other participants included regional Primary Care Trust (PCT) staff and community pharmacists who were familiar with the trial, as well as members of the research team involved in running the trial.

### Data generation

**Figure 1:**
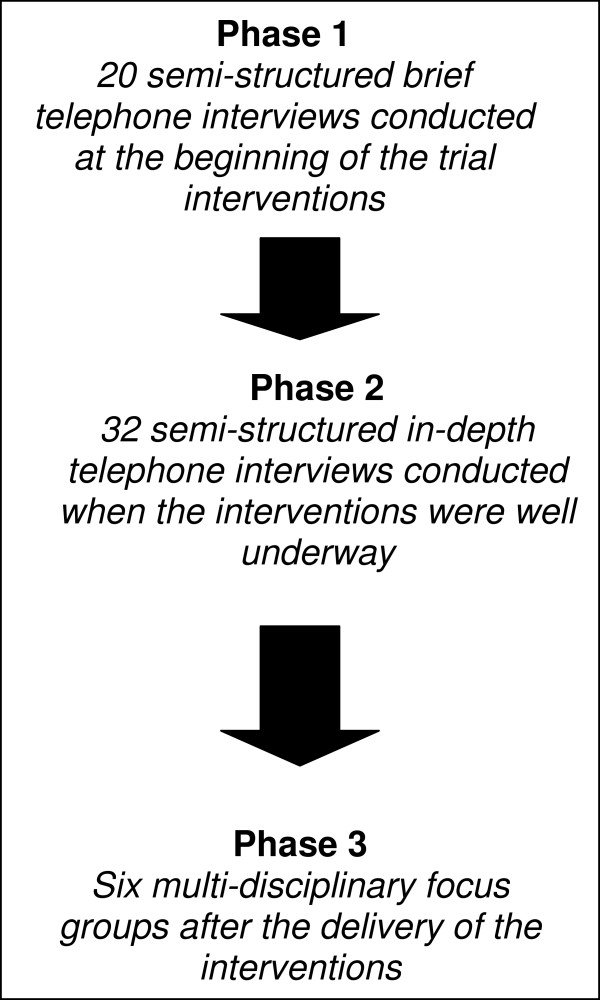
Flow diagram of the different phases of the qualitative evaluation.

A flow diagram of the study design summarizing the process of data generation and the different phases is given in Figure 1. Data were collected by a designated qualitative researcher leading on the qualitative evaluation aspect of the trial (KC).

All six PINCER pharmacists were interviewed at three different time-points during the delivery of the PINCER intervention. This included when they had just started working on the trial, when they had worked in a number of practices, and an exit interview on finishing their work on the trial [30].

Qualitative data were collected through semi-structured telephone interviews and multi-disciplinary focus groups. Focus groups were conducted in participating PINCER and Simple feedback practices. Participants in both focus groups and interviews consisted of a mix of pharmacists, GPs, practice managers, nurses, administrative staff, PCT staff, and community pharmacists. Additional data were collected from pharmacist discussion groups attended by PINCER pharmacists giving them the opportunity to exchange experiences and facilitated by the research team, notes of practice feedback meetings made by pharmacists who were part of the PINCER intervention, and diaries kept by the pharmacists over a period of 12-18 months.

Brief telephone interviews were conducted at the early stages in both arms of the trial (Figure 1: Phase 1), more in-depth interviews were conducted when both the PINCER intervention and Simple feedback were well underway (Figure 1: Phase 2) and focus groups were conducted when both the PINCER intervention and Simple feedback had been delivered (Figure 1: Phase 3).

Interviews explored general perceptions of prescribing safety in general practice, experiences and opinions of the PINCER intervention and the Simple feedback control arm, sustainability of the intervention, and expectations for the future. Core questions from the topic guides are summarized in Table 3.

**Table 3:** Core questions from interview topic guides

	
**Questions relating to the trial - experiences and opinions**	How appropriate is the trial’s design?
	What are the perceived obstacles to success?
	Are there any concerns and how could these be addressed?
	What is going well and why?
	What do participants expect in relation to outcomes?
**Questions relating to a potential roll-out of the trial**	Would this be acceptable?
	How could this be constructed?
	What could be done better?
**More general questions in relation to prescribing safety in general practice**	What are the main perceived issues?
	Can pharmacists play a valuable role in addressing these?
	How do they see the future?

Six focus groups were conducted; the main questions related to the potential future roll-out of the PINCER intervention (see Table 4).

**Table 4:** Core questions from focus group discussions

	
**The wider usability of the trial**	How might the trial interventions be modified or adapted in order to maximize their effectiveness when implemented in routine general practice?
**Possible alternative interventions**	What alternative interventions/strategies might be both acceptable to stakeholders and effective in reducing prescribing errors in general practice?
	Introduction of three potential models emerging from brief interviews including:
	Simple feedback - practices themselves setting up and conducting searches on a monthly basis
	Training practice staff to provide relevant clinical input
	Pharmacist intervention

### Data analysis

Interviews and focus groups were transcribed verbatim. Broad themes and sub-themes were identified employing thematic analysis using NVivo7 software to aid the coding process [32]. Transcripts and notes were analyzed one by one, creating themes and sub-themes until no new themes emerged. Data from meetings and diaries were checked against the emerging thematic structure. Particular attention was paid to comparing themes identified across different sources, professional groups, geographical locations and intervention/control arm. In analyzing data, we drew on key principles from the diffusion of innovations literature, which provided a valuable conceptual framework of how innovations are adopted by individuals over time and how these then spread through organizations [33,34].

The main researcher (KC) and AS met regularly to discuss emerging findings; the credibility of the data and the analysis was enhanced by close involvement and discussions with the wider research team. Results were periodically fed back to the Trial Management Group and to PINCER pharmacists in order to confirm the validity of emerging results. In addition, a second qualitative researcher (SS) re-coded brief and in-depth interview transcripts and gave thorough feedback on the preliminary analysis to the primary researcher (KC), who then incorporated this in the write-up of the work. This resulted in minor amendments to the emerging findings.

We continued with data generation until no new themes emerged. This resulted in a general framework capturing the essence of the data collected at each stage. Careful attention was paid to the comparison and integration of different professional groups and intervention/control arms (that is, PINCER intervention and Simple feedback). This was accomplished through seeking out deviant cases and actively exploring alternative explanations. Careful attention was also paid to taking a non-judgmental approach to all aspects of data generation, as the issue of prescribing errors was viewed as a potentially sensitive topic. At the time of the initial round of data collection, none of the participants (except one pharmacist) had had any contact with the researcher beforehand. It was hoped that this anonymity also facilitated disclosure of potential barriers and/or problems.

### Ethical considerations

Ethical approval was obtained from the Nottingham 2 Research Ethics Committee (Reference Number: 05/Q2404/26) and local research management approvals were obtained. Advanced disclosure and honorary contracts were obtained prior to the study. All participants gave written informed consent and had the opportunity to withdraw from the study at any time. Transcripts were anonymized and any identifiable information was kept in a locked cabinet in a locked office by the researcher collecting the data.

## Results

A full description of the qualitative dataset for each study phase can be viewed in Table 5. Overall, we collected and analyzed data from 52 semi-structured telephone interviews; six pharmacist diaries, notes from four pharmacist discussion groups, feedback meetings in 34 practices, and six focus group discussions.

**Table 5:** A description of the full qualitative dataset for each study phase

	
**Phase 1**	· 23 participants were invited to take part including all six PINCER pharmacists, seven GPs, six practice managers, two researchers and two PCT prescribing leads.
	· Three participants were excluded as they either felt unqualified to answer questions or did not return the researcher’s calls.
	· Additional data were collected from two facilitated pharmacist discussion groups at which the qualitative researcher took notes.
**Phase 2**	· 37 participants were invited to take part including all PINCER pharmacists (each interviewed twice), 11 GPs, nine practice managers, two community pharmacists, five nurses and four prescribing leads.
	· Five participants declined to participate, mainly due to time constraints.
	· Further data were collected from two audio-taped pharmacist facilitated meetings. One was facilitated by the trial coordinator with the qualitative researcher taking notes. The other was designed as an informal focus group, with the qualitative researcher facilitating and the trial coordinator taking notes.
	· Additional qualitative data consisted of notes of practices meetings made by pharmacists during the delivery of the PINCER intervention and six pharmacist diaries.
**Phase 3**	· Six focus groups were conducted with a total of 30 participants.
	· Four focus groups were with practice staff from practices in PINCER intervention and Simple feedback practices including four practice managers, one assistant practice manager, ten GPs, two nurses, two data quality officers, one administrative staff, one junior doctor, and one medical student.
	· One telephone focus group was conducted with PINCER pharmacists.
	· One focus group with PCT staff in location 2 and one interview with a member of the PCT in location 1 (including Medical Advisors, professions involved in medicines management, and those from clinical and pharmaceutical backgrounds).
	· The research team initially approached practices that were recommended by the PINCER pharmacists and those that had reduced numbers of patients in relation to the outcome measures at follow-up. However, due to a lack of willingness to participate, it was decided to widen the sampling strategy and therefore almost all practices that had participated in the trial were subsequently approached. The most commonly mentioned reason for refusing was that practices were busy and felt that they had already given up a lot of their time for the trial.

The study indicated that the PINCER intervention had considerable credibility among stakeholders. Face-to-face and regular interaction between the pharmacist and practice staff was reported to be important. However, the study also identified some important tensions relating to some of the outcome measures investigated, integration of the pharmacist into the practice team and the nature of pharmacists’ work. Participants also pointed to the important role of local health communities (conceptualized as practices in close geographical proximity to each other) in relation to any future potential roll-out. These issues are considered in detail below and are structured along the following key themes:

• Credibility of the issue being addressed and the PINCER intervention

• Regular interaction

• The nature of outcome measures and the resulting way they are addressed in practices

• Integration of pharmacists into the practice team

• The nature of pharmacists’ work

• The likely central role of local health communities in extending the PINCER intervention into routine models of care.

### Credibility of the issue being addressed and the PINCER intervention

All GPs and their teams recognized that prescribing errors were an important and potentially preventable problem and there was a widely held belief that a pharmacist-centered intervention was a credible solution.

"‘I think pharmacists are obviously much, much better informed than we are er, and although actually often maybe some of the hospital specialists would have ideas about that they can be very idiosyncratic in their ideas with things so it’s actually pharmacies [sic], Pharmacists will tend to often be a lot more evidence based in what they’re telling us and will have looked into it in a bit more detail rather than their own personal preferences. So it’s almost a more, I think it’s almost a more reliable source of information about drugs when we’re trying to make decisions about whether we’re needing to alter our practice.’"

(GP 2, PINCER intervention practice, Focus Group 1, Location 1).

"‘Erm, certainly, erm, makes us more aware of erm, one or two areas like beta-blocker, eye drops, erm, I think some of us were not aware of, that they, that there could be a problem with asthmatics for example.’"

(GP, PINCER intervention practice, Brief Interview 5, Location 1).

Pharmacists in all PINCER intervention practices were therefore highly valued and given the authority to address many of the issues identified themselves. Both the PINCER intervention and the Simple feedback control arm were viewed as minimally disruptive in the busy general practice environment, and most practices were willing, and in some cases positively inclined, towards having their databases periodically interrogated. The trade-off or relative advantage between disruption and identifying potentially serious errors was considered acceptable by the majority of practice staff.

"Interviewer: I mean would that be something that you think would be useful on a regular basis, on an on-going basis to have some sort of audit like that. I mean I would be quite interested to know whether, you know, sort of just having sort of, you know, say the searches run maybe every six months or something like that. Would that be anything that would be of interest to you?"

"GP: I think so yes. It would be of interest and I think also it’s important from a patient safety point of view so again if it’s unobtrusive and it’s a manageable workload, then yes. If it’s all set up just keep running it."

(GP 1, Simple feedback practice, Focus Group 2, Location 1).

### Regular interaction

Practice staff, and GPs in particular, believed that it was important for a designated person to drive through any changes that needed to be made (that is, the pharmacist encouraging changes to happen). In line with this, ongoing face-to-face contact between pharmacist and practice staff in PINCER intervention practices was particularly valued.

"‘But I think you can’t discount the human-human interaction you get when someone cajoles you and says, you know, says to you, ‘You really should be doing this, it’s just a little job to do it’. It’s so much better than a little email going ‘ping’. Email from automatic pharmacist in some other place, they’re somewhere else, would you please look at this. It’s just not quite the same. I don’t know if that’s, in fact does that matter, that we should be professionals, we should do it anyway but we’re all human beings and…because it isn’t just the numbers that motivate people the human interaction is also what makes people think well we ought, we really ought to do something about this.’"

(GP 2, PINCER intervention practice, Focus Group 2, Location 1).

The lack of ongoing pharmacist input may also explain the attenuation in all primary outcome measures at the 12 month assessment point (Table 2). Similarly, Simple feedback practices were not given any further support, making the implementation of changes suggested by the educational material more dependent on key individuals from within the practice to drive change. In most practices, these individuals appeared to be practice managers, who took on the responsibility for organizing the trial in the practice and driving changes forward.

"Interviewer: And your role as a Practice Manager, how would you describe that?"

"Practice Manager: Far reaching and wide ranging. And, do you mean in terms of the PINCER Trial? Co-ordinating it. From the practice point of view and liaising with the hospital…I have met with the erm, [name] and I’ve met with Professor, what’s his name? Professor [name]. I liaised with them and arranged meetings with my chief erm, my senior partner. I’ve met with the software chap that did the reports, erm and I’ve got the reports on my PC [personal computer] and I liaise with the doctors about actioning whatever has come out of those reports."

(Practice manager, Simple feedback practice, In-depth Interview 11, Location 1).

However, the busy practice environment meant that there were often conflicting priorities within practices.

"‘I think they’ve got so many priorities and in that particular case they were a single-handed practice erm, so many priorities that erm, some of the issues that, you know, with PINCER such as monitoring erm, are possibly not top of the list.’"

(Trial Pharmacist, Brief Interview 9, Location 2).

### The nature of outcome measures and the resulting way they are addressed in practices

The biggest reductions in the number of errors were in relation to failures of drug monitoring (see Table 2). This may have been due to pharmacists helping practices to establish more effective mechanisms of communication between primary and secondary care, which has repeatedly been recognized as an important problem in both the United Kingdom and worldwide [35,36].

"‘I think what we really should be taking forward is this shared care which is quite a worry, you know, the hospital are doing one thing and we’re doing another and we’re supposed to be sharing the care. We’re prescribing the medication but we’re not getting to know the results from the hospital.’"

(Nurse, PINCER intervention, In-depth Interview 10, Location 1).

Nevertheless, we also identified some tensions across practices that may have impacted on effectiveness. For example, not all ‘errors’ were necessarily seen as failings by clinicians. A particularly prominent example here was in relation to patients with a history of asthma being prescribed beta-blockers. Although this outcome was significantly reduced at both six and 12 months, many GPs believed that individualized patient-based risk assessments needed to be made. This led GPs in some instances to continue prescribing the beta-blocker, over-ruling pharmacists’ advice on the basis of clinical experience and in-depth knowledge of the individual patient.

"‘GPs felt it was difficult to comment without knowing the identity of the patients and their history. The issue of risk/benefit for patients with CHD (coronary heart disease) was raised. Also discussion around patients identified as having ‘history of asthma’ which may have arisen when notes were summarised based on e.g. a single prescription many years ago for a salbutamol inhaler prescribed for a severe chest infection.’ (From pharmacist feedback sessions)."

### Integration of pharmacists into the practice team

Another source of concern related to pharmacists’ sense of isolation, as in many cases they felt they were not effectively integrated into the practice team. The relatively short time period (three days per week for up to 12 weeks) spent in individual practices was stated to be a key contributory factor to this difficulty in developing and sustaining meaningful clinical relationships by all PINCER pharmacists. In addition, the location of the pharmacist while undertaking work within the PINCER intervention practices was raised as a concern by some and contributed to feelings of isolation in these individuals.

"‘Erm, it can be a little bit lonely being the only pharmacist working in a GP practice for, you know, a couple of days. Erm, especially when you’re only there temporarily. If you were there permanently and you were really, really were part of their team, part of the payroll then it would be different. But because you are only there twelve weeks you don’t fully integrate into their team. Erm, you know, it’s hard, I mean when we come to work, we all come to work for money obviously but, you know, you also want to build relationships with the people that you work with and when you’re moving on to somewhere new every twelve weeks you don’t get that element of it I suppose.’ (Trial Pharmacist, Brief Interview 8, Location 2)."

Recognizing this issue, the research team arranged regular trial pharmacist discussion groups giving pharmacists the opportunity to exchange experiences. However, although this was found to help reduce feelings of isolation, other contributing factors were more difficult to address; for example, the way pharmacists were accommodated in the practices. Most pharmacists repeatedly reported having to work in busy locations such as the reception area, move desks several times a day, not having an identified work station or having to work in a room remote from the main practice.

"‘Some days there has been nowhere at all for me to work and I have had to work in the tearoom – difficult as people are coming in and out and there is no computer access.’ (Pharmacist diary, Pharmacist 2)"

"‘I had very little to do with GPs/nurses. Was given a room in the attic, so did not see anyone during the day, unless I came out of my room to make tea/coffee etc.’ (Pharmacist diary, Pharmacist 6)"

### The nature of pharmacists’ work

All PINCER pharmacists also raised issues with work satisfaction. These included reports of missing direct patient contact and a feeling that their work for the trial was frustrating at times as often problems encountered in practices were beyond the scope of their trial work. There was also a general concern among most that the delivery of the same standardized PINCER intervention was repetitive and not offering the desired flexibility of tailoring outcomes and interventions to meet either their own needs or the broader needs of participating practices. In addition, the perceived uncertainty surrounding job security with pharmacists being employed on temporary contracts was viewed as inhibiting a more stable and (importantly) long-term relationship between pharmacists and practice staff.

"‘I don’t think people left the job because they felt it boring, I think it was circumstantial that, and you know, with the best will in the world however exciting this job was, and I don’t think it’s the most exciting job I’ve ever had but it’s certainly not awful, I don’t think any of us have thought it was awful at all. I think sometimes it can be a bit monotonous but any job is monotonous, I mean I’d be saying exactly the same practice work can be monotonous, so don’t know, sort of unless you’re having patient contact and that was just never going to be feasible in this study, I think it was just, because it’s just contracts and, you know, you’ve got to have a job when this ends and unfortunately you can’t always time it that it happens exactly when you want it to.’ (Pharmacist 1, Focus Group)."

### The likely central role of local health communities in extending the PINCER intervention into routine models of care

We developed insights into how the PINCER intervention could be incorporated into routine practice, with most practice staff and pharmacists suggesting that PINCER pharmacists’ work could be incorporated into a primary care pharmacist role. Currently, most PCTs employ pharmacists to work with selected practices in relation to medicines management, and participants believed that the outcome measure searches and change management activities could be incorporated into this work. However, with the abolition of PCTs in England by April 2013, primary care commissioning responsibility is being transferred to the newly formed general practice clusters known as Clinical Commissioning Groups [37]. These are increasingly responsible for commissioning services of primary care pharmacists and, therefore, in terms of potential roll-out of the PINCER intervention, it would be the Clinical Commissioning Groups that would be employing pharmacists to work with their cluster practices.

Although some drawbacks were acknowledged and the extent of involvement may need further consideration, the primary care pharmacist model was advocated repeatedly in focus groups as it would allow for adequate leadership, skill-base and support/training structures. Such a model may also help to address some of the issues with work satisfaction raised by PINCER pharmacists.

In line with the perceived general importance of incentives, the recommendation to use the Quality and Outcomes Framework (QOF) was also proposed by some GPs [38].

"GP 1: I’m just wondering whether you could link it to QOF because, you know, in the QOF there’s a specific indicator around is it one or two or three prescribing, three prescribing initiatives… And whether or not you could link one of those initiatives to, in particular to a patient safety aspect of prescribing… Might be a way to start and creep with it. (…)"

"Interviewer: …So can I ask you now which one would you choose after discussing, or if you can think of any more please erm, let us know but er, which one would you go for?"

"GP 1: If we had to mainstream it and we were really serious about making it work then I think I would go with the PCT involvement model."

"Practice Manager: Link it to QOF think that would be a wonderful way to take it forward."

"GP 1: Yeah."

(Simple feedback practice, Focus Group 2, Location 1)

Pharmacists recommended that, in order to allow for more flexibility, pharmacists’ involvement could be cluster based (or in the future Clinical Commissioning Group-based), with one pharmacist looking after a group of practices. This fits in well with the national drive to give local health communities more autonomy [39].

### Interpreting data in the light of the existing theoretical literature

Both Roger’s work on the diffusion of innovation and Greenhalgh’s work on the diffusion of innovation in health service organizations proved helpful in interpreting our findings [33,34]. These frameworks helped to conceptualize the importance of perceived innovation attributes and the role of key individuals in facilitating diffusion of the PINCER intervention (conceptualized as an innovation) across healthcare organizations.

The perceived value of the PINCER intervention can be viewed in terms of what Rogers refers to as ‘relative advantage’ and ‘compatibility’ [34]. Relative advantage, is the extent to which the innovation is seen as better than current practice. Compatibility is the extent of agreement between the innovation and individual (or organizational) values and beliefs. Patient safety is clearly an organizational priority and optimal levels are an aspiration, making the trial interventions compatible. However, in order for interventions to be compatible they also need to be acceptable to both individuals and settings where they are implemented (in this case GP practices) and those delivering them (in this case pharmacists). In the healthcare setting, studies investigating the predictive power of these two innovation attributes and adoption behavior have been mixed [40-42]. Although some have suggested that diffusion of innovations in healthcare is slow if it involves acquiring new skills [40], our work clearly supports the view that adoption is more influenced by the perceived improvements in patient safety [42].

"‘…you find out something and we are doing it and know intentionally it will be a good thing to know that, why we shouldn’t be doing that. (…) Yeah, well it will be effective because you are flagging the patients out who’ve been prescribed perhaps inappropriately or unintentionally the things they shouldn’t prescribe where they can cause more complications to them. (…) What will I, will be actually alert, you know, next time we prescribe other things…’"

(GP, Simple feedback practice, Brief Interview 6, Location 2)

PINCER pharmacists may be viewed as ‘change agents’. In the diffusion of innovations literature, these are individuals who influence clients’ innovation decisions in a direction deemed desirable by a ‘change agency’ [34]. It is the change agency’s aim to implement the innovation with a focus on the collective goals of the social system (here to improve prescribing safety). Our findings support the notion of the important role of pharmacists as change agents, as well as the importance of a good personal relationship and ongoing face-to-face contact between change agents and clients (that is, practice staff) in facilitating adoption of innovations in primary care settings [33,34,43-45].

GPs are local ‘opinion leaders’ [46]. Their involvement is therefore clearly important for the successful adoption of innovations as any apprehensiveness on their part in this respect clearly has a high chance of influencing other members of the practice team [46-48]. Although Rogers argued that ideally the change agent should harness the influence of opinion leaders, the nature of this relationship remains under-researched [34]. In our trial, the pharmacist as the change agent had the potential to influence opinion leaders, but this was to a large extent dependent on an effective working relationship between the pharmacist and the GP. If this was not the case, and this crucial relationship is characterized by infrequent contact, power struggles, or opinion leaders’ resistance to the change agent’s efforts, it could serve to act as a major barrier to the diffusion of the innovation. Such relationship considerations may have contributed to pharmacists reporting experiences of difficulties in getting GPs to, on occasions, make prescribing changes.

Practice managers frequently played the role of a ‘change aide’ in working with the pharmacist to facilitate the interventions. This was often expressed in terms of providing a link between the pharmacist and GPs and helping the pharmacist to engage more with GPs so that they would complete actions forms. Therefore, systematically harnessing the practice manager’s influence might contribute to maximizing the impact of the PINCER intervention.

"‘Well if it’s something related to the surgery or practice or my working then I ask the practice manager, he like, some of the doctors, what happened was I left some of them envelopes with some action plans in them. When I went back the next week they were still there, the doctors hadn’t picked them up or some of them were a bit lazy picking their work out of the pigeon holes and so basically the practice manager said, ‘I’ll sort it out, don’t worry’. Then he’s going to go and nag them now with the envelopes.’"

(Trial Pharmacist, In-depth Interview 6, Location 2).

## Discussion

The PINCER Trial represents one of the world’s first successful efforts at clearly establishing the effectiveness of a complex pharmacist-led information technology-enabled intervention, aiming to improve prescribing safety in primary care [18,49,50]. This embedded longitudinal evaluation has provided important insights into why the PINCER intervention was so effective [51,52]. Nevertheless, some trends of improvement were also observed in Simple feedback practices, highlighting the important role of motivational issues in initiating and sustaining change. Important contributory factors included the GPs’ perceived importance of the clinical interventions and the perceived appropriateness of a pharmacist-based approach to address this issue. Central to this was ongoing face-to-face contact between pharmacists and practice staff. However, pharmacists also expressed some concerns about the likely sustainability of the pharmacist intervention model in the absence of an appropriate support network and career development pathways for pharmacists. Incorporating the PINCER pharmacists’ role into routine local primary care pharmacists’ work may provide a solution to these issues. Our work indicates that this could for example be taken forward by Clinical Commissioning Groups employing pharmacists to work with their cluster practices performing outcome measure searches and change management activities routinely.

### Strengths and potential limitations of the present study

This embedded qualitative evaluation has generated an in-depth nuanced account of the underlying processes of the interventions under investigation. Data were longitudinally collected from a range of sources (participants and geographical locations), through a variety of data collection methods (interviews, documents including diaries, and focus groups), thereby allowing us to trace developments over time and develop personal relationships with participants, which possibly allowed franker discussions than might have otherwise been the case [53,54]. The longitudinal nature of the inquiry enabled us to gain insights into how changes were initiated and sustained over time, helping to take into account the likely role of initial enthusiasm for initiating changes combined with the everyday reality of sustaining them. Similarly, pharmacists’ accounts over time revealed that although initially PINCER intervention work was viewed as interesting, there was, with time, a degree of monotony associated with the role. Trustworthiness of the study was increased through a second qualitative researcher examining the interview transcripts and reports, as well as having constant input from the extended research team.

Although participating practices were larger and more likely to be training practices than non-participating practices, they were not atypical in any key respects. Similarly, PINCER pharmacists came from a variety of backgrounds (some with primary care experience and some without, some with higher degrees and some without) and seemed to be representative of the general practice pharmacist population.

However, there are also some limitations to our study. Practices are encouraged to improve prescribing safety through the QOF, which is part of the General Medical Services National Health Service Contract introduced in April 2004 [38]. This framework includes a list of indicators of practice performance that determine practice payments with the help of a point system. One of these indicators relates to the quality and safety of prescribing. This may have influenced our results in both PINCER intervention and Simple feedback control arms as practices may have addressed the issues identified as being motivated by the framework rather than by the PINCER intervention. Alternatively, framework indicators may take priority over trial outcome measures as the framework is closely related to payment incentives.

Our study has supported the value of telephone interviews in reaching geographically scattered individuals with busy work schedules [55,56]. However, the most distinct disadvantage of telephone interviews is that the interviewer cannot respond to non-verbal cues of participants. Also, questions are more easily misunderstood on the telephone. Conversely, the anonymity of this method of data collection might have facilitated disclosure about a sensitive topic such as prescribing and medication errors.

The focus groups clearly complemented the interviews, mainly because it was possible to gain a deeper understanding of the group dynamics and a range of (potentially differing) viewpoints among participants [57]. On the other hand, participants in the focus groups may have been inhibited in expressing their personal opinion due to the presence of others. Therefore, it is common practice in qualitative methods to use interviews and focus groups in combination [58].

### Implications for policy, practice and research

The findings from this work raise the question of whether there is now need to implement more widespread use of pharmacists in the primary care setting to reduce potentially preventable medication errors. We have provided a starting point for the conceptualization of this model and outlined some issues that would need to be addressed to maximize its effectiveness and acceptability among key stakeholders. We summarize potential areas for further work based on our findings in Table 6.

**Table 6:** Potential questions for future research

	
**Questions**	How can pharmacists (or other healthcare professionals) be integrated more efficiently into established care teams? How can multi-disciplinary collaboration be more effective?
	What other effective incentives may be viable to exploit in primary care (other than financial incentives)?
	How can audit and feedback techniques be refined to include feedback of the impact of the interventions?
	How can a more positive and close relationship between pharmacists and GPs be fostered?
	How can we design interventions that target all key players and a range of practice staff?
	Which intervention types are appropriate for which practices? Is effectiveness influenced by practice characteristics?
	How can macro issues be addressed, particularly communication issues with secondary care and nursing homes and polypharmacy?
	How exactly may the suggested community-based pharmacist model be implemented into routine care? To what extend can we allow for flexibility in this context?

## Conclusions

Our study clearly supports the value of qualitative evaluation methods in complementing randomized controlled trials of complex interventions. Overall, the interventions were delivered as planned and stakeholders perceived them as valuable. This was particularly so in the PINCER intervention arm. There was also a distinct feeling that pharmacist intervention could be incorporated into the routine work of practice pharmacists employed by the National Health Service to reduce prescribing errors.

The most important factor distinguishing this pharmacist intervention from other interventions to reduce clinically important errors in medicines management in general practice appeared to be the face-to-face contact with practice staff and the designated role of the pharmacist as a change agent. Diffusion of Innovation Theory provided a useful theoretical background and has helped to integrate the current findings with the existing literature.

## Competing interests

SS was employed as a trial pharmacist but got involved in the present towards the end of the study, when data from all pharmacists were already collected. The other authors declare that they have no competing interests.

## Authors’ contributions

AS, TA, SAM and JC conceived this qualitative evaluation. KC led recruitment, supported by SR, and undertook data generation (also supported by SR in relation to the focus groups) and analysis. SS supported in data validation and helped with recruitment and some aspects of data collection (a practice focus group). AA was the Chief Investigator for the PINCER study and AS was the Principal Investigator overseeing the qualitative work. KC and AS led the drafting of the manuscript, and all authors commented on draft papers. KC and AS are guarantors. All authors read and approved the final manuscript.
